# PP38 Assessment Outcome By Type Of Available Evidence: Retrospective Analysis Of Oncological Early Benefit Assessment Reports

**DOI:** 10.1017/S0266462324002101

**Published:** 2025-01-07

**Authors:** Jonas Goretzko, Annette Pusch-Klein, Beate Wieseler

## Abstract

**Introduction:**

For early benefit assessment in Germany, drugs must be assessed at market entry as to whether they provide an added benefit versus the current standard therapy. This retrospective analysis focuses on assessment reports prepared by the independent Institute for Quality and Efficiency in Health Care (IQWiG). The objective was to describe the relationship between available evidence and assessment outcome.

**Methods:**

Data were retrospectively extracted from IQWiG’s early benefit assessment reports of oncological drugs that entered the market between June 2021 and December 2021. The underlying evidence for the benefit assessments was divided into three groups: direct comparison (based on a randomized controlled trial [RCT]), indirect comparison, and other sources (e. g., only a single-arm trial was submitted). Afterwards, the outcome of the assessment was described by type of available evidence.

**Results:**

In the period under review, IQWiG worked on 27 oncology projects addressing 40 different oncological research questions resulting in 46 conclusions. About half of the available evidence in the benefit assessments was a direct comparison with standard therapy based on an RCT (n=22/46). From this level of evidence, an added benefit was derived for the drug under assessment in 64 percent of cases. Indirect comparisons (n=7/46) mostly led to an added benefit not proven (86%). If only other sources were submitted (n=17/46), the added benefit was assessed as not proven in all projects examined.

**Conclusions:**

This retrospective analysis highlights the pivotal role of RCTs in providing strong evidence for health technology assessment. Conversely, single-arm and observational studies fail to yield substantial evidence. In the future, RCTs should be made simpler, faster, and cheaper; for example, by integrating them into existing infrastructures in routine practice (e.g., registry-based RCTs) or by conducting randomized adaptive platform trials.

